# A questionnaire measure of adult attachment anxiety correlates with frontal hemispheric asymmetry in sleep spindle activity

**DOI:** 10.1007/s41105-022-00426-0

**Published:** 2022-10-20

**Authors:** Melinda Becske, Imre Lázár, Róbert Bódizs

**Affiliations:** 1 Department of Psychiatry and Psychotherapy, Semmelweis University, Budapest, Hungary; 2 Institute of Behavioural Sciences, Semmelweis University, Budapest, Hungary; 3 Institute of Social and Communication Sciences, Károli Gáspár University of the Reformed Church in Hungary, Budapest, Hungary

**Keywords:** Frontal laterality, Slow sleep spindles, Emotional reactivity, Attachment anxiety, Neuroticism

## Abstract

Subjects with high levels of attachment anxiety and neuroticism were proposed to be characterized by higher relative right rather than left frontal activity. Since sleep spindles are argued to reflect enhanced offline neuroplasticity, higher spindle activity measured over the right frontal areas relative to the corresponding left frontal ones could index higher attachment anxiety and neuroticism. Our aim was to explore the relationship between the lateralization patterns of frontally dominant slow sleep spindles and questionnaire measures of adult attachment anxiety and neuroticism. Thirty-four healthy subjects (male = 19; *M*_age_ = 31.64; SD_age_ = 9.5) were enrolled in our preliminary study. Second night EEG/polysomnography records and questionnaire measures of personality (Zuckerman–Kuhlman Personality Questionnaire) and adult attachment (Relationship Scales Questionnaire) were collected. Frontal slow sleep spindles were measured by the Individual Adjustment Method (IAM), whereas hemispheric asymmetry indexes of spindle occurrence rate, duration, and amplitude were derived as normalized left–right differences (electrode pairs: Fp1–Fp2, F3–F4, and F7–F8). Relative right lateralization of frontolateral and frontopolar slow sleep spindle density and mid-frontal slow spindle duration were associated with attachment anxiety, but spindle lateralization was less closely related to neuroticism. The relationships between frontal slow spindle laterality and attachment anxiety remained statistically significant even after controlling for the effect of neuroticism, whereas attachment avoidance–independence was not correlated with frontal slow spindle lateralization. Right frontal lateralization of slow sleep spindle activity might indicate attachment status in terms of the negative view of the self.

**Supplementary Information:**

The online version contains supplementary material available at 10.1007/s41105-022-00426-0.

## Introduction

Attachment, as a psychological term introduced by Bowlby, refers to an inborn, biologically based behavioural system that ensures survival of the offspring [1]. Approach and withdrawal mechanisms drive the behaviour of infants: they tend to move away from objects causing distress and approach their caregivers for comfort [1]. Individual differences exist in the attachment behaviour, that is referred to as secure and insecure (avoidant or anxious-ambivalent) attachment style [2]. Avoidant children tend to be more engaged with the environment and less likely to seek proximity of their parents, while ambivalent children seem clingy on their parents and less engaged with the environment [2].

Attachment style and emotional regulation are closely related even in adulthood [3]. Adults with secure attachment—having a basic feeling of trust and competence—develop better coping strategies, they are emotionally more flexible. It has been argued that they are more willing to explore their own emotions, and can better regulate them [3]. Attachment insecurity, on the other hand, is considered to be a risk factor for developing mental and physical health problems [2]. Insecure attachment style hinders social competence and the ability to form and maintain close relationships. Attachment insecurity can have devastating consequences on the individuals’ lives by causing them to develop early maladaptive schemas [4].

Previous research suggest that frontal asymmetry is related to emotional–motivational processes both on trait and state level [5]. According to the revised reinforcement sensitivity theory, three basic systems regulate human behaviour that are mostly stable from early childhood throughout the lifetime [6]: Behavioural Activation System (related to anticipated positive affect of goal attainment, reward responsiveness and extroversion [7], found to be mediated by left prefrontal areas [8]), Fight–Flight–Freeze System (fear, negative affect: related to subcortical structures [9]), and a Supervisory control system (revised Behavioural Inhibition System [r-BIS]: related to punishment, nonreward, behavioural inhibition, and risk analysis, control of different motivational urges [6], mediated by right prefrontal areas [9]). The idea of this kind of functional differentiation between the two hemispheres, regarding emotional–motivational processing, is strongly supported by empirical data [including lesion studies (see for review, [9])]. Lateralization of prefrontal activity is found to be at least partly driven by the dopaminergic system [10], and it is closely tied to reward processing [11] and reward anticipation [12].

R-BIS is related to effortful control, self-control, the urge to act in a socially desirable manner [9]. This system controls behaviour by increasing sensitivity towards negative stimuli [6]. Empirical results suggest that higher resting state left to right frontal asymmetry positively correlates with mental health; e.g., Mikolajczak and colleagues [13] found positive correlation between emotional intelligence and left frontal asymmetry, in a study with 31 healthy participants. Relative left frontal dominance during rest correlated with less difficulty in everyday emotional regulation (impulse control) on a sample of 80 adults [14].

Individual differences in these basic systems controlling behaviour are regarded stable from early childhood through the lifetime. Functioning of these systems is partly predetermined by genetic factors (e.g., polymorphisms of dopaminergic and serotonergic receptor genes [15]) and is also shaped during childhood in the attachment relationship [16].

Regarding attachment style, a considerable amount of literature has dealt with the question of emotional processing biases related to attachment anxiety—and also with related constructs, e.g., anxiety, social insecurities, social threat, and neuroticism—in general, and in terms of frontal hemispheric asymmetry as well [17–19]. For example, Gartstein and colleagues [20] investigated changes in the laterality patterns of infants (*N* = 50 infant–mother pairs) during a mildly stressful task. According to their results, higher left prefrontal activation correlated with more positive affectivity, more synchronous and reciprocal interactions between infants and mothers. They concluded that left prefrontal asymmetry was associated with better emotional control.

Few researchers have addressed the question of frontal asymmetry during sleep so far, and the studies found mixed results [21–23]. Researchers have only investigated hemispheric laterality as a function of alpha electroencephalogram (EEG) asymmetry, assuming that “alpha” reflects cortical inactivity both in wakefulness and during sleep [21]. However, as frequency characteristics of sleep spindling—in fact known to be a marker of cortical reactivation rather than inhibition [24]—overlap with the alpha range, we think that the question of frontal laterality during sleep has to be re-investigated from a different perspective.

Sleep spindles are burst-like oscillations at the intersection of alpha and beta range occurring during NREM sleep [25]. Researchers distinguish slow (frontally dominant) and fast (centro-parietally located) spindles [25]. Spindling activity is related to neural plasticity and synaptic potentiation: region-specific reactivation of brain circuits, enhanced neuroplasticity that facilitates offline memory consolidation, and system-level reorganization within functional networks [24].

Evidence suggest that localized spindling activity correlates with improvement of highly lateralized skills (e.g., motor memory [26]). Empirical data also suggest that baseline lateralization patterns of sleep spindles are age- and gender-specific, assumed to reflect the functional and anatomical differences in the brain [27]. However, little is known about the relationship of trait-like differences in lateralization of spindling activity and cognitive–emotional dispositions: lateralized psychological functions.

Since lateralization of sleep spindles was found to correlate with asymmetric involvement of hemispheres during online information processing in case of memory tasks [26], we assume that it can also apply to certain trait-like dispositions in cognitive–emotional functioning that appear to show specific lateralization patterns, as well [8, 17, 28, 29]. We hypothesize that relative left domination of frontal slow spindle lateralization correlates negatively with psychological traits that are argued to be related to enhanced right or reduced left hemispheric frontal activity, namely attachment anxiety and trait-like emotional reactivity indexed by neuroticism.

## Materials and methods

### Participants

Overall 34 healthy adults participated in the study (male = 19; *M*_age_ = 31.64; SD_age_ = 9.5; Med_age_ = 29.5; IQR_age_ = 13); however, participants with extreme scores [abs(z-score) > 3] were excluded from the respective analyses on a pairwise basis. More data on participants and subject recruitment can be found in the Supplementary material.

### Polysomnographic recordings

Sleep data were recorded for two consecutive nights in the laboratory by standard polysomnography, including EEG according to the 10–20 system. EEG was recorded form electrodes: Fp1, Fp2, F3, F4, Fz, F7, F8, C3, C4, Cz, T3, T4, T5, T6, P3, P4, O1, and O2. In addition, a bipolar EOG derived from contacts above and below the left and the right canthi, submental EMG, ECG, and breathing (thoracic and one abdominal respiratory inductive plethysmography) were measured. The sampling rate of the amplifier was 249 Hz. EEG data were re-referenced offline to the mathematically linked mastoids [(A1 + A2)/2]. Sleep EEG recordings of the second nights spent in the laboratory were analyzed. EEG data were manually scored on a 20 s basis by applying standard criteria [30]. Artifacted epochs were removed on the basis of visual inspection on a 4 s basis.

### Psychometric data

Dimensions of attachment (anxiety and avoidance–independence) were measured by the Hungarian adaptation of the Relationship Scales Questionnaire (RSQ-HUN, [31]). The questionnaire is based on Bartholomew’s two-dimensional model of adult attachment [32]. For measuring neuroticism, we administered the Hungarian adaptation of the Zuckerman–Kuhlman–Aluja Questionnaire (ZKPQ-HUN, [33]). The questionnaire has a strong biopsychological basis. Items of the neuroticism subscale measure nervousness, worry, anxiety, insecurity, and sensitivity. Both are self-report questionnaires.

### EEG data analysis

Spindle activity of all-night NREM sleep was examined with the IAM approach described in previous studies [34]. Frequency criteria of slow and fast spindles were based on the individual-specific peaks of spectra between 9 and 16 Hz (upper and lower frequencies of individual spectral peaks were determined by zero-crossing points of the averaged second-order derivatives of the amplitude spectra). Sleep spindle frequencies (fast and slow) were tested for centro-parietal and frontal dominance, respectively. The amplitude criteria were determined in individual-and derivation-specific manner by multiplying the number of intra-spindle frequency bins with the mean amplitude spectrum values corresponding to lower and upper frequency limits. EEG was then band-pass filtered—based on the FFT method—for individual slow and fast sleep spindle frequencies. Envelopes of the filtered signals were calculated. EEG segments meeting the amplitude criteria lasting for at least 0.5 s were considered spindles. Based on the IAM approach, individual- and derivation-specific densities (spindles × min^−1^), durations (s), and amplitudes (μV) of slow and fast spindles were calculated.

### Statistics

Since we followed a hypothesis-driven approach, and we were particularly interested in frontal asymmetry during NREM sleep, we chose to examine slow spindle laterality, as slow sleep spindles are known to show a frontal dominance [35]. Laterality index of slow spindle activity (spindle density, duration, and amplitude) was computed with the following equation (laterality index > 0 indicate greater left lateralization, while laterality index < 0 indicate greater right lateralization of sleep spindles):

1 $${\rm spindle\, laterality\, index} = ({\rm left} - {\rm right})/({\rm left} + {\rm right}).$$

Because of the small sample size and violation of normal distribution, correlations and partial correlations between variables were computed with Spearman’s rank correlation coefficient. To correct for the effect of multiple testing, the Benjamini–Hochberg correction was performed.

## Results

**Fig. 1:**
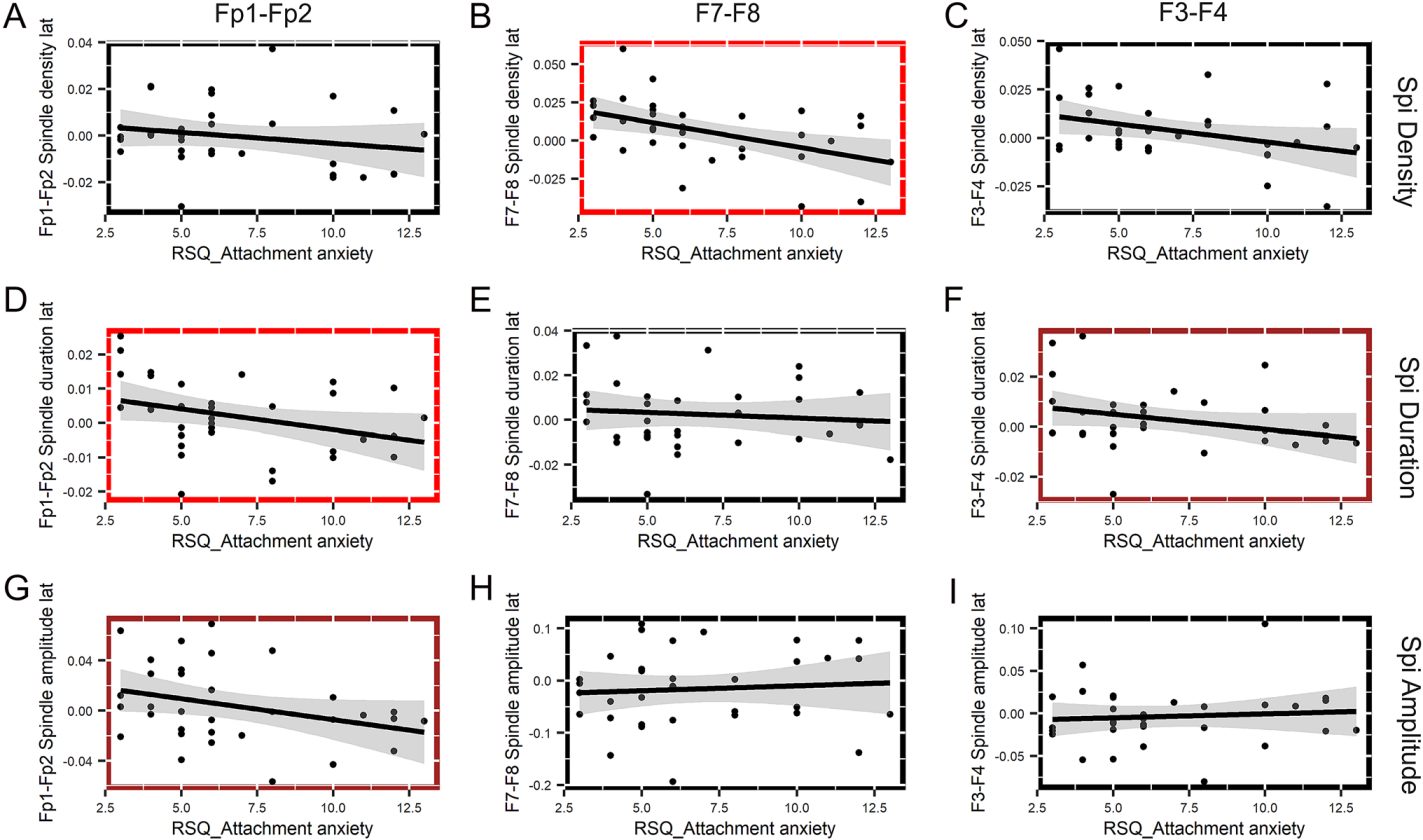
Correlations between attachment anxiety and frontal slow sleep spindle lateralization. Subplots show the relationship between attachment anxiety (*x*-axis) and different measures of frontal slow spindle activity (density, duration, and amplitude) at each frontal electrode pair (Fp1–Fp2, F7–F8, and F3–F4). The linear relationship between RSQ attachment anxiety and slow sleep spindle laterality is indicated by regression lines and their confidence intervals, whereas statistical significance was tested by Spearman coefficients. Statistically significant correlations (*p* < 0.05) are indicated with red, and trend-level correlation (*p* < 0.1) is indicated with brown

Features of frontal slow spindle lateralization correlated with one out of the two measured attachment dimensions. Attachment anxiety (*M* = 6.765, SD = 3.006) was related to relative right frontal slow spindle dominance: i.e., the higher a subject scored on the attachment anxiety dimension, the more right lateralized his/her frontal slow spindles were. This was reflected by the correlations between attachment anxiety and the lateralization of slow spindle density in the lateral frontal and frontopolar electrode sites (*r*_s AttAnx_vs_F7–F8 (SpiDens)_ = − 0.506, *p* = 0.002; *r*_s AttAnx_vs_Fp1–Fp2 (SpiDur)_ = − 0.410, *p* = 0.016). Attachment anxiety was related to the lateralization of spindle duration in the mid-frontal area (*r*_s AttAnx_vs_F3–F4 (SpiDur)_ = − 0.340, *p* = 0.053) and to the lateralization of spindle amplitude in the frontopolar area (*r*_s AttAnx_vs_Fp1–Fp2 (SpiAmp)_ = − 0.333, *p* = 0.058) on a trend level only. Attachment anxiety did not correlate with the other measures of frontal slow spindle laterality (Fig. 1, Table 1).

**Table 1:**
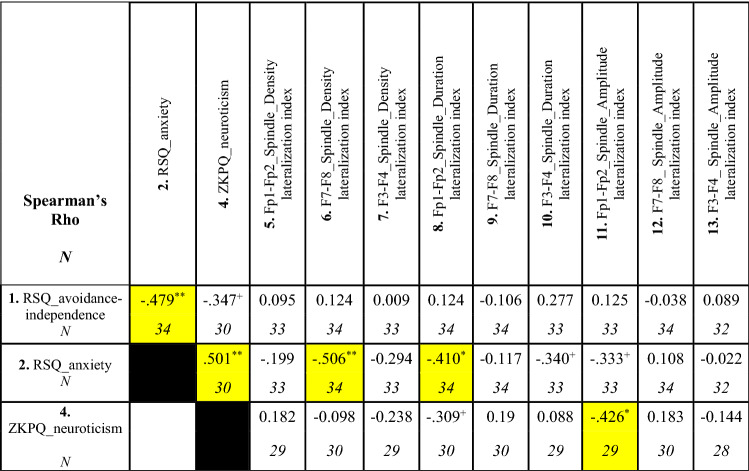
Correlations between frontal slow sleep spindle lateralization and psychometric variables (Spearman’s rho)

Table 1 shows the results of rank correlational analyses. Statistically significant correlations are highlighted in yellow

^+^*p* < 0.1, ^*^p < 0.05, ^**^p < 0.01

**Fig. 2:**
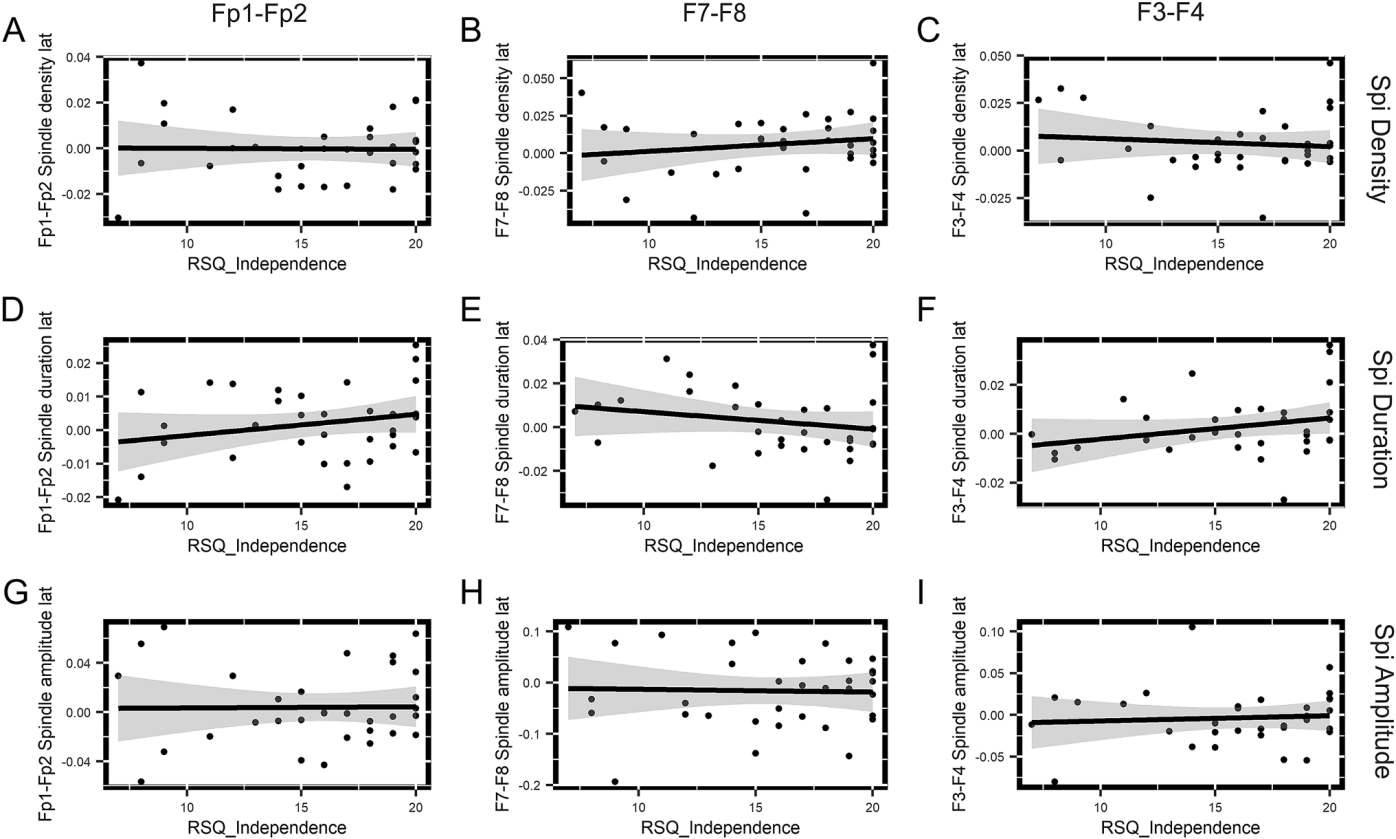
Correlations between attachment avoidance–independence and frontal slow sleep spindle lateralization. Subplots show the relationship between attachment avoidance–independence (*x*-axis) and different measures of frontal slow spindle activity (density, duration, and amplitude) at each frontal electrode pair (Fp1–Fp2, F7–F8, and F3–F4). The linear relationship between RSQ attachment anxiety and slow sleep spindle laterality is indicated by regression lines and their confidence intervals, whereas statistical significance was tested by Spearman coefficients

Correlations between attachment avoidance–independence (*M* = 15.618, SD = 4.053) and frontal slow spindle lateralization were not statistically significant (Fig. 2, Table 1). After performing the Benjamini–Hochberg procedure, all of the significant correlations remained statistically significant at a false discovery rate of 25%.

**Fig. 3:**
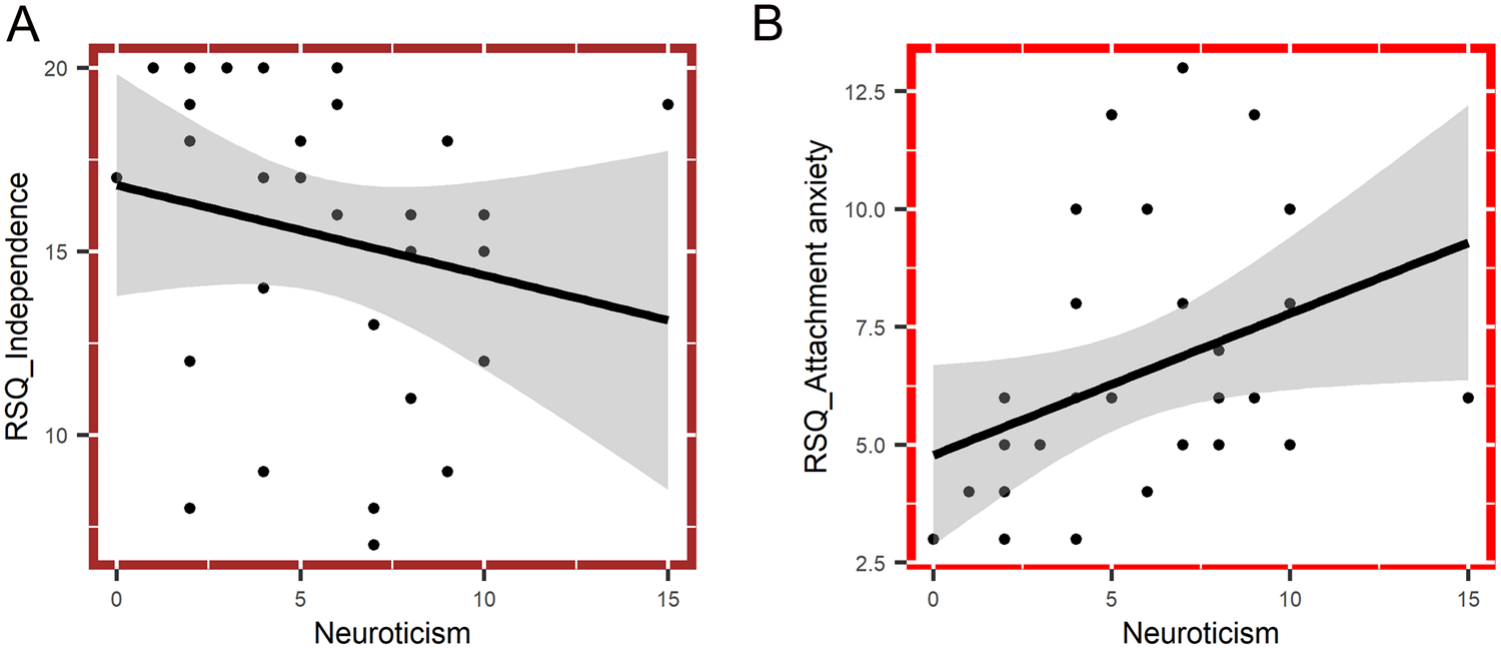
Correlations between psychometric variables. Subplots show the relationship between psychometric variables. The linear relationship between RSQ attachment anxiety and slow sleep spindle laterality is indicated by regression lines and their confidence intervals, whereas statistical significance was tested by Spearman coefficients. Statistically significant correlations (*p* < 0.05) are indicated with red, and trend-level correlation (*p* < 0.1) is indicated with brown

Neuroticism (*M* = 5.6, SD = 3.43) positively correlates with attachment anxiety (*r*_s Neur_vs_AttAnx_ = 0.501; *p* < 0.01). The correlation between neuroticism and attachment avoidance–independence failed to reach statistical significance, it was only significant on a trend level (*r*_s Neur_vs_AttInd_ = − 0.347; *p* < 0.1; Fig. 3).

**Fig. 4:**
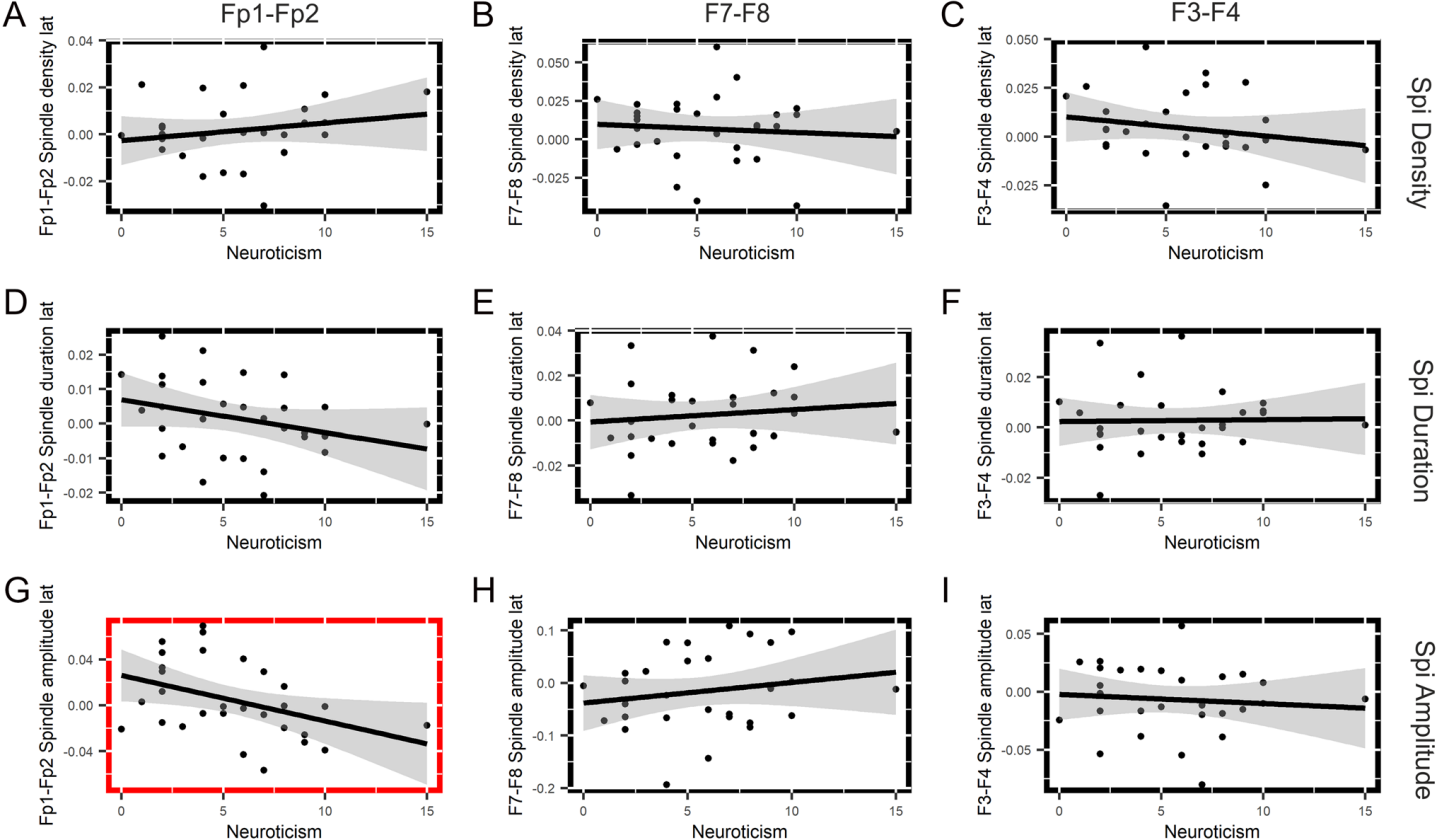
Correlations between neuroticism and frontal slow sleep spindle lateralization. Subplots show the relationship between neuroticism (*x*-axis) and different measures of frontal slow spindle activity (density, duration, and amplitude) at each frontal electrode pair (Fp1–Fp2, F7–F8, and F3–F4). The linear relationship between RSQ attachment anxiety and slow sleep spindle laterality is indicated by regression lines and their confidence intervals, whereas statistical significance was tested by Spearman coefficients. Statistically significant correlations (*p* < 0.05) are indicated with red

Neuroticism was negatively correlated with relative left lateralization of slow sleep spindle amplitude in the frontopolar electrode sites (*r*_s Neur_vs_Fp1–Fp2 (SpiAmp)_ = − 0.426, *p* = 0.021), but it was not related to the other measures of spindle lateralization at any location (Fig. 4, Table 1).

Although attachment anxiety positively correlated with neuroticism [*r*_s AttAnx_vs_Neur_ = 0.501; *p* < 0.01 Table 1)], most of the above mentioned correlations remained statistically significant even after controlling for the effect of neuroticism. Attachment anxiety was clearly related to relative right rather than left frontal slow spindle dominance [lateral frontal spindle density: *r*_s AttAnx_vs_F7–F8 (SpiDens)_ = − 0.603; *p* = 0.0005; frontopolar spindle duration: *r*_*s* AttAnx_vs_Fp1–Fp2 (SpiDur)_ = − 0.427; *p* = 0.02; mid-frontal spindle duration *r*_s AttAnx_vs_F3–F4 (SpiDur)_ = − 0.557; *p* = 0.002 (Table 2)].

**Table 2:**
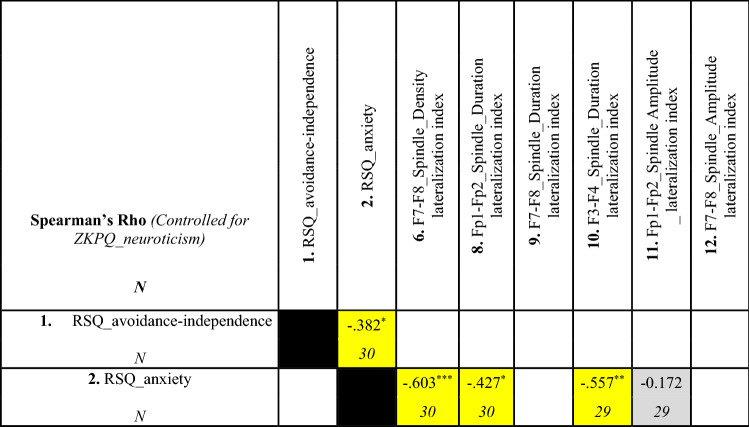
Partial correlations between spindle lateralization and psychometric variables (Spearman’s rho, after controlling for the effect of neuroticism)

Table 2 shows the results of the rank correlational analyses, where the effect of neuroticism is partialled out. Statistically significant correlations are highlighted in yellow

^*^p < 0.05, ^**^p < 0.01, ^***^p < 0.001

## Discussion

In the present study, we aimed to investigate the association between attachment anxiety and the lateralization of frontal slow sleep spindles, examining three features of spindling activity: density, duration, and amplitude. Based on the assumptions that (1) regional increase in spindling activity during NREM sleep indicates reactivation of brain areas more actively engaged in neurocognitive functioning at wakefulness [26, 27], and (2) individual differences in baseline spindle lateralization exist [27], we hypothesized that adult attachment anxiety would be inversely related to relative left dominance of frontal slow spindling activity.

The main results of this study indicate that attachment anxiety and relative frontal slow sleep spindle laterality are related indeed, in accordance with our hypothesis, as we found a negative relationship between attachment anxiety score and relative left lateralization of frontal slow sleep spindles in terms of spindle density in the lateral frontal (F7–F8) and frontopolar (Fp1–Fp2) areas. Furthermore, lateralization of spindle duration in the mid-frontal (F3–F4) area and lateralization of spindle amplitude in the frontopolar (Fp1–Fp2) area correlated with the attachment anxiety score on a trend level. This can indicate an overactive right and/or underactive left frontal hemisphere, leading to the over-activation of the Behavioural Inhibition System. That causes an elevated level of trait negative affect, anxiety, increased risk analysis and self-control, heightened sensitivity towards negative stimuli, and lower ability to control negative emotional impulses [9].

As mentioned above, only a few studies examined the association between frontal asymmetry during sleep and state or trait affect. Sikka and colleagues [22] investigated the relation of alpha asymmetry and state-like dream affect, and found a relationship between dream anger and decreased activity in the right relative to the left frontal hemisphere, based on alpha asymmetry during REM sleep and evening resting wakefulness. Their results can be related to diminished ability to regulate intense emotions or negatively valenced approach-motivational urges during dreaming, as a function of weaker relative right lateralization of frontal cortical activity. Schmidt and colleagues [21], on the other hand, analyzed the association between frontal alpha asymmetry during sleep and a questionnaire measure of trait motivational style (Behavioural Inhibition Scale). The authors found a moderately strong, negative correlation between the BIS questionnaire score and relative alpha power in the left frontal hemisphere during stage two sleep. As greater relative alpha power in the right frontal area would indicate relative left dominance in wakefulness, the authors regarded this result as contradictory. However, remembering that frequency characteristics of alpha waves and slow spindles overlap, this result can be straightforwardly interpreted on the ground of current evidence on the function of sleep spindles [24, 26]. The other dimension of the attachment questionnaire, i.e., attachment avoidance–independence did not seem to correlate with the lateralization of slow spindling activity.

Since, previous research suggests that both attachment issues and laterality of frontal activity can be related to the personality dimension of neuroticism, we tested whether the above relationships remain statistically significant after controlling for the effect of neuroticism. We found that each of the statistically significant correlations remained statistically significant even after partialling out the linear effect of neuroticism. In addition, the trend-level correlation between attachment anxiety and lateralization of slow spindle amplitude in the frontopolar (Fp1–Fp2) area vanished after controlling for neuroticism, but the correlation of attachment anxiety and the lateralization of spindle duration in the mid-frontal (F3–F4) area turned out to be statistically significant only after partialling out the effect of neuroticism. Regarding the relations between neuroticism and adult attachment, neuroticism was found to be positively related to both attachment anxiety and the need for close relationship, but its negative relationship with attachment avoidance–independence was only significant on a trend level. Contrary to our expectations, neuroticism did not turn out to be closely related to frontal slow spindle lateralization, as it was only significantly correlated to the lateralization of spindle amplitude at the frontopolar (Fp1–Fp2) area.

Lateralization indexes derived from the three features of spindling activity (density, duration, and amplitude) correlated differently with questionnaire data: the anxiety dimension correlated with the lateralization of spindle density (in case of one electrode pair) and duration (in one electrode pair), while neuroticism was related to the lateralization of spindle amplitude (in one electrode pair). Based on current knowledge on the subject, it is hard to give an adequate interpretation of this aspect of the results. Furthermore, it also has to be admitted that correlations were not significant at every frontal electrode pair analyzed.

Further research with larger sample sizes and more refined research protocols—e.g., gathering more data regarding daytime events to take any possible confounding factors into account—would be necessary to confirm the link between certain psychological traits and hemispheric lateralization of sleep spindles.

Although this is a pilot study, the findings suggest that examining the relations between certain psychological factors and lateralization patterns of sleep spindles can be a useful way of gaining more knowledge concerning the relationship of personality and brain activity. As relations of psychological measures and frontal asymmetry are normally estimated based on small amounts of EEG data (e.g., few minutes long resting state recordings), analysis of whole night sleep recordings offers an opportunity to gain more robust results (based on more data). Future research might consider investigating the question, whether and how the different features of sleep spindling activity—and the underlying mechanisms behind them—can be specifically related to certain psychological factors, e.g., neuroticism or different dimensions of adult attachment (on larger samples, of course). In addition, more comprehensive and sophisticated statistical methods (e.g., mediation and moderation analyses) could be applied to test more complex relations between variables.

Future studies could include clinical samples with mood- and attachment-related issues as well. Potential therapy-related changes in frontal lateralization patterns could also be investigated during wakefulness and sleep.

## Conclusion

Lateralization of frontal slow sleep spindle activity was found to be weak-to-moderately related to a questionnaire measure of adult attachment: attachment anxiety. The major significance of the study is that it proposes a novel approach to the analysis of sleep EEG data in terms of hemispheric lateralization of cortical activity which is found to be related to trait-like emotional dispositions.

### Supplementary Information

Below is the link to the electronic supplementary material.

Supplementary file1 (DOCX 15 KB)

## Data Availability

The data that support the findings of this study are available from the corresponding author upon reasonable request.
