# Supplementary material for: A questionnaire measure of adult attachment anxiety correlates with frontal hemispheric asymmetry in sleep spindle activity
Source: Sleep Biol Rhythms. 2022 Oct 20;21(2):155–63. doi: 10.1007/s41105-022-00426-0 (PMC10899928; doi:10.1007/s41105-022-00426-0)
Supplement: Supplementary file 1 — Supplementary file1 (DOCX 15 KB) [file 41105_2022_426_MOESM1_ESM.docx]

**1. Participants**

Convenience and snowball sampling methods were applied to recruit subjects. Questionnaires were used to screen for sleep disorders, and semi-structured interviews were carried out by experienced psychiatrists or psychologists to assess subjects’ mental health. According to the interviews all subjects were healthy, had no history of neurologic or psychiatric disease (including mood disorders of course), and were free of any current drug effects. Consumption of alcohol was prohibited, but subjects were allowed to consume a maximum of two cups of coffee until noon on the days of sleep EEG-registration.

Although, we did not gather detailed information regarding the subjects’ daytime activities or mood states, they were requested to avoid any significant changes in their daytime routine, or any events causing extreme psychological or physical stress, as well as to report if such events occurred.

**2. Sleep macrostructure data**

|  | RSQ_Independence | RSQ_anixiety | ZKPQ_Neuroticism |
| --- | --- | --- | --- |
| RSQ_Independence | 1 | 1 | 1 |
|  | - | - | - |
| Sleep_duration (min) | **-0.341** | **0.322** | **0.495** |
|  | **0.049** | **0.063** | **0.005** |
| Sleep_efficiency (%) | -0.069 | 0.103 | 0.197 |
|  | 0.699 | 0.562 | 0.296 |
| Sleep_latency (min) | -0.199 | 0.156 | 0.040 |
|  | 0.258 | 0.379 | 0.833 |
| WASO_after_first_not_S1_sleep (min) | 0.086 | -0.100 | -0.069 |
|  | 0.629 | 0.572 | 0.717 |
| REM_latency (min) | 0.232 | -0.190 | -0.089 |
|  | 0.187 | 0.281 | 0.639 |
| Wake_duration (min) | -0.071 | 0.020 | -0.015 |
|  | 0.691 | 0.911 | 0.936 |
| Relative_wake_duration (%) | 0.072 | -0.119 | -0.192 |
|  | 0.685 | 0.504 | 0.309 |
| N1_duration (min) | **-0.326** | 0.034 | 0.182 |
|  | **0.059** | 0.847 | 0.337 |
| Relative_N1_duration (%) | -0.173 | -0.045 | 0.068 |
|  | 0.328 | 0.801 | 0.721 |
| N2_duration (min) | -0.189 | 0.106 | 0.315 |
|  | 0.284 | 0.551 | 0.090 |
| Relative_N2_duration (%) | 0.078 | -0.194 | -0.074 |
|  | 0.661 | 0.272 | 0.699 |
| N3_duration (min) | 0.060 | 0.071 | 0.097 |
|  | 0.735 | 0.690 | 0.610 |
| Relative_N3_duration (%) | 0.204 | -0.082 | -0.102 |
|  | 0.248 | 0.644 | 0.590 |
| REM_duration (min) | **-0.345** | **0.461** | **0.475** |
|  | **0.046** | **0.006** | **0.008** |
| Relative_REM_duration (%) | -0.224 | **0.426** | **0.320** |
|  | 0.203 | **0.012** | **0.085** |

**Table 1** Correlations between sleep macrostructure data and psychometric variables
